# 5,7-Bis(benz­yloxy)-2-phenyl-4*H*-chromen-4-one

**DOI:** 10.1107/S160053680803938X

**Published:** 2008-11-29

**Authors:** Angannan Nallasivam, Munirathinam Nethaji, Nagarajan Vembu, Buckle Jaswant, Nagarajan Sulochana

**Affiliations:** a Department of Chemistry, National Institute of Technology, Tiruchirappalli 620 015, India; b Department of Inorganic and Physical Chemistry, Indian Institute of Science, Bangalore 560 012, India; c Department of Chemistry, Urumu Dhanalakshmi College, Tiruchirappalli 620 019, India; d Department of Chemistry, Government Arts College, Karur 639 005, India

## Abstract

In the title compound, C_29_H_22_O_4_, the chromene ring is almost planar with a small puckering [0.143 (2) Å]. The crystal structure is stabilized by C—H⋯O and C—H⋯π inter­actions. Edge-to-face (centroid–centroid distances of 3.894 and 3.673 Å) and face-to-face (centroid–centroid distance of 3.460 Å) π–π-ring electron inter­actions are also observed.

## Related literature

For the biological and pharmacological properties of benzopyrans and their derivatives, see: Brooks (1998 ▶); Hatakeyama *et al.* (1988 ▶); Hyana & Saimoto (1987 ▶); Tang *et al.* (2007 ▶). For the importance of 4*H*-chromenes, see Liu *et al.* (2007 ▶); Wang, Fang *et al.* (2003 ▶); Wang, Zhang *et al.* (2003 ▶). For hydrogen-bond motifs, see: Bernstein *et al.* (1995 ▶); Desiraju (1989 ▶); Desiraju & Steiner (1999 ▶); Etter (1990 ▶).
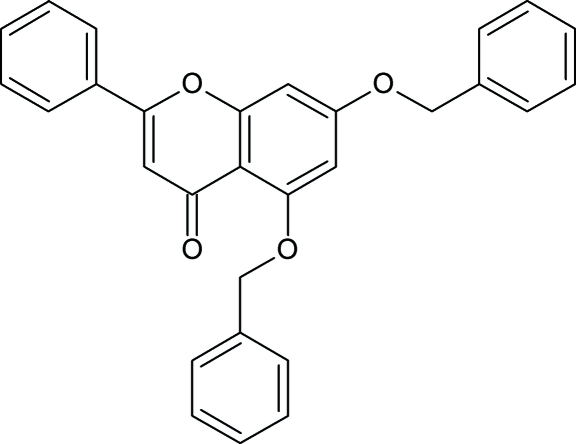


## Experimental

### 

#### Crystal data

C_29_H_22_O_4_
*M* *_r_* = 434.47
Triclinic, 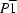

*a* = 9.496 (3) Å
*b* = 11.572 (3) Å
*c* = 11.767 (3) Å
α = 66.564 (4)°
β = 79.668 (5)°
γ = 73.836 (5)°
*V* = 1136.1 (5) Å^3^
*Z* = 2
Mo *K*α radiation
μ = 0.08 mm^−1^
*T* = 293 (2) K
0.45 × 0.33 × 0.23 mm

#### Data collection

Bruker SMART APEX CCD diffractometer
Absorption correction: multi-scan (*SADABS*; Sheldrick, 1998 ▶) *T*_min_ = 0.963, *T*_max_ = 0.981
13435 measured reflections
5302 independent reflections
3534 reflections with *I* > 2σ(*I*)
*R*_int_ = 0.018

#### Refinement

*R*[*F* ^2^ > 2σ(*F* ^2^)] = 0.059
*wR*(*F* ^2^) = 0.155
*S* = 1.05
5302 reflections
298 parameters
H-atom parameters constrained
Δρ_max_ = 0.20 e Å^−3^
Δρ_min_ = −0.23 e Å^−3^

### 

Data collection: *SMART* (Bruker, 2007 ▶); cell refinement: *SAINT* (Bruker, 2007 ▶); data reduction: *SAINT*; program(s) used to solve structure: *SHELXS97* (Sheldrick, 2008 ▶); program(s) used to refine structure: *SHELXL97* (Sheldrick, 2008 ▶); molecular graphics: *PLATON* (Spek, 2003 ▶); software used to prepare material for publication: *SHELXL97*.

## Supplementary Material

Crystal structure: contains datablocks I, global. DOI: 10.1107/S160053680803938X/fb2125sup1.cif

Structure factors: contains datablocks I. DOI: 10.1107/S160053680803938X/fb2125Isup2.hkl

Additional supplementary materials:  crystallographic information; 3D view; checkCIF report

## Figures and Tables

**Table 1:** Hydrogen-bond geometry (Å, °)

*D*—H⋯*A*	*D*—H	H⋯*A*	*D*⋯*A*	*D*—H⋯*A*
C16—H16⋯O17^i^	0.93	2.57	3.212 (3)	127
C30—H30⋯*Cg*1^ii^	0.93	3.12	3.838	135
C8—H8⋯*Cg*2^iii^	0.93	3.29	4.066	142
C27—H27*B*⋯*Cg*2^iii^	0.97	3.18	4.083	156

Symmetry codes: (i) 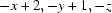; (ii) 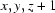; (iii) 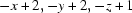. *Cg*1 and *Cg*2 are the centroids of the C20–C25 and C28–C33 rings, respectively.

**Fig. 1.**
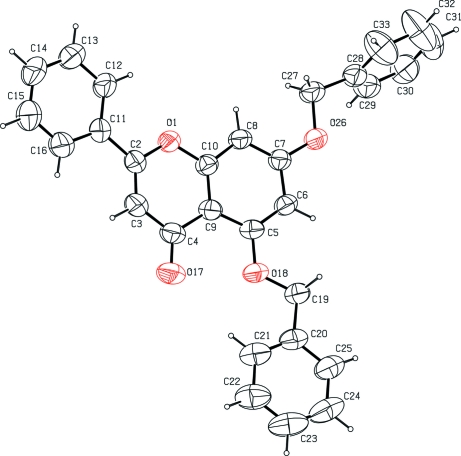
The title molecule showing the displacement ellipsoids depicted at the 50% probability level for all non-H atoms. The hydrogen atoms are drawn as spheres of arbitrary radius.

**Fig. 2.**
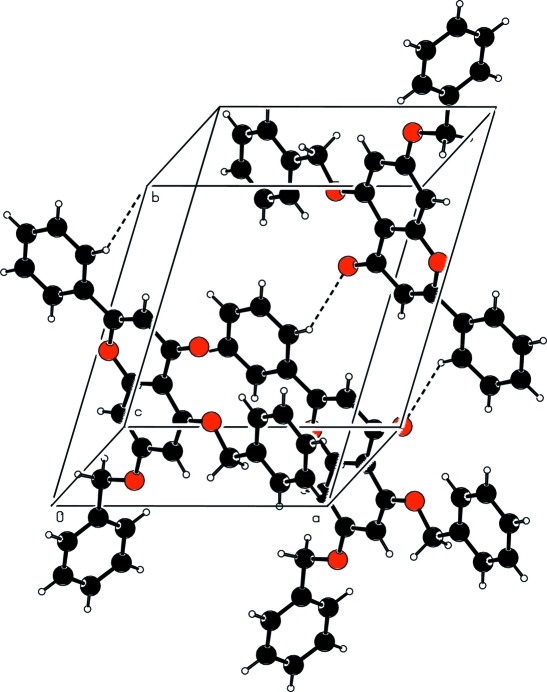
The molecular packing viewed down the a-axis. Dashed lines represent weak C–H···O interactions.

## supplementary crystallographic information

### Comment

Chromenes (benzopyrans) and their derivatives have numerous biological and pharmacological properties (Tang *et al.*, 2007) such as antisterility (Brooks, 1998) and anticancer activity (Hyana & Saimoto, 1987). In addition, polyfunctionalized chromene units are present in numerous natural products (Hatakeyama *et al.*, 1988). 4*H*-chromenes are important synthons for some natural products (Liu *et al.*, 2007). As a part of our structural investigations on 4*H*-chromene derivatives and compounds containing the benzopyran fragment, the single-crystal X-ray diffraction study on the title compound was carried out.

The chromene ring is almost planar similarly as those found in the related chromene derivatives (Wang, Zhang *et al.*, 2003; Wang, Fang *et al.*, 2003). The total puckering amplitude of the chromene ring is 0.143 (2) Å in the title structure. The interplanar angle between the chromene ring and the 2-phenyl ring is 6.8 (2)° thereby indicating the almost coplanar arrangement (Fig. 1). The benzyl group at C5 is slightly distorted from coplanarity with the chromene ring whereas the benzyl group at C7 is clearly non-coplanar as discerned from the respective interplanar angles of 7.6 (1)° and 70.01 (7)°.

The crystal structure is stabilized by the interplay of C–H···O and C–H···π interactions (Fig. 2, Table 1; Desiraju, 1989; Desiraju & Steiner, 1999). The C12–H12···O1 interaction is involved in a motif of a graph set S(5) (Bernstein *et al.*, 1995; Etter, 1990). In another S(5) motif, C21–H21···O18 interaction is involved. The C8–H8···*Cg*2^ii^ and C27–H27···*Cg*2^ii^ (*Cg*2 is the centroid of the ring C28\C29···C33) interactions take part in the motif of the graph set *R*^1^_2_(7) where the entire *Cg*2 ring C28\C29···C33 is considered as a single acceptor atom.

There are two edge-to-face π···π interactions between *Cg*3 (O1\C2\C3\C4\C9\C10) and *Cg*4 (C5\C6\C7\C8\C9\C10) [2-*x*, 2-*y*, -*z*] at 3.894 Å with α = 4.44, β = 26.68, γ = 31.06° and perpendicular distances being 3.336 and 3.480 Å, *Cg*4 and *Cg*1 (C20\C21\C22\C23\C24\C25) [1-*x*, 2-*y*, -*z*] at 3.673 Å with α = 6.87, β = 20.69, γ = 16.31° and perpendicular distances being 3.525 and 3.436 Å. There is a face to face π···π interaction between two symmetery related *Cg*4 (2-*x*, 2-*y*, -*z*) rings at 3.460 Å with α = 0.00, β = 10.24, γ = 10.24° and perpendicular distances being 3.405 Å (α is the dihedral angle between the planes I and J where I is the plane of centroid 1 and J is the plane of centroid 2, β is the angle between the vector Cg(I)→Cg(J) and the normal to plane I, γ is the angle between the vector Cg(I)→ Cg(J) and the normal to plane J, the two perpendicular distances denote the perpendicular distances of Cg(I) on ring J and Cg(J) on ring I).

### Experimental

A suspension of chrysin (3.93 mmol, 1.00 g) and potassium carbonate (11.81 mmol, 1.64 g) in dimethyl formamide (10 ml) were added into a round bottom flask. The reaction mixture was heated to 383 K for 2–3 h. The reaction mixture was then cooled to 353 K and benzyl chloride (15.74 mmol, 1.99 g) was slowly added to the reaction mixture with the help of a dropping funnel. The reaction mixture was maintained for 8–9 h at 353 K and monitored by a high pressure liquid chromatography (HPLC). After completion of the reaction, the content was quenched with water and stirred for 30–45 min at 303 K. The obtained crude solid was filtered and washed with plenty of water followed by methanol and dried under vacuum at 343 K. The crude product was dissolved in 20 ml of 1:1 (volume) mixture of dichloromethane and n-hexane. The clear solution was kept for a week without stirring. Diffraction quality prism shaped crystals of average size 0.3 mm were obtained which were filtered and washed with n-hexane and dried under vacuum at 343 K. Yield: 90%

### Refinement

All the H-atoms were observed in the difference electron density map. However, they were situated into idealized positions with C–H = 0.93 and 0.97 Å for aryl and methylene H, respectively, and constrained to ride on their parent atoms with *U*_iso_(H)=1.2Ueq(C).

### Crystal data

**Table:**

C_29_H_22_O_4_	*Z* = 2
*M**_r_* = 434.47	*F*_000_ = 456
Triclinic, *P*1	*D*_x_ = 1.270 Mg m^−^^3^
Hall symbol: -P 1	Melting point = 439–441 K
*a* = 9.496 (3) Å	Mo *K*α radiation λ = 0.71073 Å
*b* = 11.572 (3) Å	Cell parameters from 589 reflections
*c* = 11.767 (3) Å	θ = 2.5–27.5º
α = 66.564 (4)º	µ = 0.08 mm^−^^1^
β = 79.668 (5)º	*T* = 293 (2) K
γ = 73.836 (5)º	Prism, colourless
*V* = 1136.1 (5) Å^3^	0.45 × 0.33 × 0.23 mm

### Data collection

**Table:**

Bruker SMART APEX CCD diffractometer	5302 independent reflections
Radiation source: fine-focus sealed tube	3534 reflections with *I* > 2σ(*I*)
Monochromator: graphite	*R*_int_ = 0.018
Detector resolution: 0.3 pixels mm^-1^	θ_max_ = 28.0º
*T* = 293(2) K	θ_min_ = 1.9º
ω scans	*h* = −12→12
Absorption correction: multi-scan(SADABS; Sheldrick, 1998)	*k* = −15→15
*T*_min_ = 0.963, *T*_max_ = 0.981	*l* = −15→15
13435 measured reflections	

### Refinement

**Table:**

Refinement on *F*^2^	Secondary atom site location: difference Fourier map
Least-squares matrix: full	Hydrogen site location: difference Fourier map
*R*[*F*^2^ > 2σ(*F*^2^)] = 0.059	H-atom parameters constrained
*wR*(*F*^2^) = 0.155	*w* = 1/[σ^2^(*F*_o_^2^) + (0.0655*P*)^2^ + 0.1818*P*] where *P* = (*F*_o_^2^ + 2*F*_c_^2^)/3
*S* = 1.05	(Δ/σ)_max_ < 0.001
5302 reflections	Δρ_max_ = 0.20 e Å^−^^3^
298 parameters	Δρ_min_ = −0.23 e Å^−^^3^
88 constraints	Extinction correction: none
Primary atom site location: structure-invariant direct methods	

### Special details

**Table:**

Geometry. All e.s.d.'s (except the e.s.d. in the dihedral angle between two l.s. planes) are estimated using the full covariance matrix. The cell e.s.d.'s are taken into account individually in the estimation of e.s.d.'s in distances, angles and torsion angles; correlations between e.s.d.'s in cell parameters are only used when they are defined by crystal symmetry. An approximate (isotropic) treatment of cell e.s.d.'s is used for estimating e.s.d.'s involving l.s. planes.
Refinement. Refinement of *F*^2^ against ALL reflections. The weighted *R*-factor *wR* and goodness of fit *S* are based on *F*^2^, conventional *R*-factors *R* are based on *F*, with *F* set to zero for negative *F*^2^. The threshold expression of *F*^2^ > σ(*F*^2^) is used only for calculating *R*-factors(gt) *etc*. and is not relevant to the choice of reflections for refinement. *R*-factors based on *F*^2^ are statistically about twice as large as those based on *F*, and *R*- factors based on ALL data will be even larger.

### Fractional atomic coordinates and isotropic or equivalent isotropic displacement parameters (Å^2^)

**Table:**

	*x*	*y*	*z*	*U*_iso_*/*U*_eq_	
O1	1.10569 (12)	0.71663 (11)	0.18466 (11)	0.0536 (3)	
C2	1.13210 (18)	0.63546 (16)	0.12145 (16)	0.0511 (4)	
C3	1.0413 (2)	0.65365 (18)	0.03827 (18)	0.0619 (5)	
H3	1.0625	0.5959	−0.0026	0.074*	
C4	0.91202 (19)	0.75798 (18)	0.00843 (17)	0.0585 (5)	
C5	0.78763 (16)	0.96910 (16)	0.04245 (15)	0.0487 (4)	
C6	0.77971 (17)	1.05076 (16)	0.10297 (15)	0.0502 (4)	
H6	0.7084	1.1279	0.0852	0.060*	
C7	0.87887 (17)	1.01797 (16)	0.19122 (15)	0.0483 (4)	
C8	0.98609 (17)	0.90462 (16)	0.21876 (15)	0.0503 (4)	
H8	1.0521	0.8825	0.2777	0.060*	
C9	0.89526 (17)	0.85064 (16)	0.06822 (15)	0.0482 (4)	
C10	0.99182 (16)	0.82506 (15)	0.15545 (15)	0.0469 (4)	
C11	1.26652 (19)	0.53363 (16)	0.15672 (17)	0.0547 (4)	
C12	1.3574 (2)	0.53208 (19)	0.23695 (19)	0.0690 (5)	
H12	1.3321	0.5957	0.2711	0.083*	
C13	1.4850 (3)	0.4377 (2)	0.2670 (2)	0.0883 (7)	
H13	1.5451	0.4385	0.3209	0.106*	
C14	1.5238 (3)	0.3431 (2)	0.2185 (3)	0.0958 (8)	
H14	1.6105	0.2799	0.2381	0.115*	
C15	1.4336 (3)	0.3424 (2)	0.1410 (3)	0.1085 (10)	
H15	1.4585	0.2773	0.1087	0.130*	
C16	1.3067 (2)	0.4365 (2)	0.1098 (2)	0.0867 (7)	
H16	1.2470	0.4346	0.0562	0.104*	
O17	0.82679 (16)	0.76574 (15)	−0.06285 (15)	0.0858 (5)	
O18	0.69794 (12)	0.99537 (12)	−0.04619 (11)	0.0609 (3)	
C19	0.59954 (19)	1.11810 (18)	−0.08836 (17)	0.0587 (5)	
H19A	0.6522	1.1861	−0.1123	0.070*	
H19B	0.5245	1.1272	−0.0227	0.070*	
C20	0.52982 (18)	1.12834 (19)	−0.19821 (16)	0.0588 (5)	
C21	0.5695 (2)	1.0332 (2)	−0.24758 (18)	0.0691 (5)	
H21	0.6430	0.9601	−0.2137	0.083*	
C22	0.5008 (3)	1.0456 (3)	−0.3475 (2)	0.0871 (7)	
H22	0.5285	0.9806	−0.3801	0.105*	
C23	0.3932 (3)	1.1517 (3)	−0.3984 (2)	0.0995 (8)	
H23	0.3478	1.1597	−0.4657	0.119*	
C24	0.3522 (3)	1.2469 (3)	−0.3498 (2)	0.0956 (8)	
H24	0.2784	1.3196	−0.3843	0.115*	
C25	0.4196 (2)	1.2359 (2)	−0.2496 (2)	0.0767 (6)	
H25	0.3907	1.3009	−0.2170	0.092*	
O26	0.86159 (12)	1.10706 (11)	0.24369 (11)	0.0595 (3)	
C27	0.9671 (2)	1.08276 (19)	0.32789 (18)	0.0655 (5)	
H27A	1.0648	1.0779	0.2855	0.079*	
H27B	0.9663	1.0009	0.3963	0.079*	
C28	0.9288 (2)	1.19031 (17)	0.37608 (16)	0.0578 (5)	
C29	0.8056 (2)	1.2047 (2)	0.45477 (18)	0.0678 (5)	
H29	0.7427	1.1492	0.4750	0.081*	
C30	0.7731 (3)	1.2992 (2)	0.5043 (2)	0.0807 (6)	
H30	0.6885	1.3082	0.5569	0.097*	
C31	0.8657 (4)	1.3795 (2)	0.4758 (2)	0.1003 (9)	
H31	0.8448	1.4434	0.5095	0.120*	
C32	0.9893 (4)	1.3666 (3)	0.3978 (3)	0.1228 (12)	
H32	1.0527	1.4214	0.3787	0.147*	
C33	1.0199 (3)	1.2724 (3)	0.3475 (2)	0.0965 (8)	
H33	1.1034	1.2647	0.2936	0.116*	

### Atomic displacement parameters (Å^2^)

**Table:**

	*U*^11^	*U*^22^	*U*^33^	*U*^12^	*U*^13^	*U*^23^
O1	0.0515 (6)	0.0537 (7)	0.0603 (7)	0.0017 (5)	−0.0168 (5)	−0.0296 (6)
C2	0.0536 (9)	0.0488 (9)	0.0564 (10)	−0.0114 (7)	−0.0058 (8)	−0.0247 (8)
C3	0.0644 (11)	0.0605 (11)	0.0732 (12)	−0.0072 (9)	−0.0160 (9)	−0.0375 (10)
C4	0.0571 (10)	0.0673 (11)	0.0617 (11)	−0.0133 (9)	−0.0150 (8)	−0.0309 (9)
C5	0.0392 (8)	0.0589 (10)	0.0505 (9)	−0.0107 (7)	−0.0094 (7)	−0.0207 (8)
C6	0.0413 (8)	0.0527 (9)	0.0542 (10)	−0.0030 (7)	−0.0119 (7)	−0.0189 (8)
C7	0.0452 (8)	0.0530 (9)	0.0508 (9)	−0.0063 (7)	−0.0084 (7)	−0.0246 (8)
C8	0.0461 (9)	0.0573 (10)	0.0523 (9)	−0.0024 (7)	−0.0154 (7)	−0.0265 (8)
C9	0.0433 (8)	0.0562 (10)	0.0500 (9)	−0.0111 (7)	−0.0063 (7)	−0.0236 (8)
C10	0.0417 (8)	0.0505 (9)	0.0489 (9)	−0.0060 (7)	−0.0066 (7)	−0.0203 (7)
C11	0.0560 (10)	0.0471 (9)	0.0635 (11)	−0.0069 (8)	−0.0085 (8)	−0.0246 (8)
C12	0.0745 (12)	0.0592 (11)	0.0775 (13)	0.0086 (9)	−0.0264 (10)	−0.0371 (10)
C13	0.0869 (15)	0.0769 (14)	0.1058 (18)	0.0158 (12)	−0.0460 (14)	−0.0452 (14)
C14	0.0860 (16)	0.0741 (15)	0.129 (2)	0.0242 (12)	−0.0414 (15)	−0.0530 (15)
C15	0.1037 (18)	0.0862 (17)	0.159 (3)	0.0276 (14)	−0.0466 (18)	−0.0866 (18)
C16	0.0804 (14)	0.0795 (15)	0.1213 (19)	0.0102 (12)	−0.0371 (13)	−0.0655 (14)
O17	0.0821 (10)	0.0934 (11)	0.1075 (11)	0.0004 (8)	−0.0444 (9)	−0.0614 (9)
O18	0.0536 (7)	0.0666 (8)	0.0682 (8)	−0.0024 (6)	−0.0269 (6)	−0.0290 (6)
C19	0.0521 (10)	0.0597 (11)	0.0642 (11)	−0.0109 (8)	−0.0187 (8)	−0.0178 (9)
C20	0.0453 (9)	0.0740 (12)	0.0528 (10)	−0.0185 (9)	−0.0099 (8)	−0.0130 (9)
C21	0.0544 (11)	0.0946 (15)	0.0583 (11)	−0.0131 (10)	−0.0102 (9)	−0.0284 (11)
C22	0.0783 (14)	0.127 (2)	0.0646 (13)	−0.0213 (14)	−0.0128 (11)	−0.0418 (14)
C23	0.0856 (17)	0.147 (3)	0.0628 (14)	−0.0260 (17)	−0.0273 (12)	−0.0263 (16)
C24	0.0728 (15)	0.108 (2)	0.0810 (16)	−0.0075 (13)	−0.0355 (12)	−0.0054 (15)
C25	0.0621 (12)	0.0810 (14)	0.0764 (14)	−0.0089 (10)	−0.0246 (10)	−0.0151 (11)
O26	0.0566 (7)	0.0597 (7)	0.0690 (8)	0.0068 (6)	−0.0251 (6)	−0.0357 (6)
C27	0.0673 (11)	0.0692 (12)	0.0670 (12)	0.0059 (9)	−0.0303 (9)	−0.0360 (10)
C28	0.0663 (11)	0.0582 (11)	0.0517 (10)	−0.0029 (9)	−0.0181 (9)	−0.0249 (9)
C29	0.0647 (12)	0.0765 (13)	0.0680 (12)	−0.0101 (10)	−0.0132 (10)	−0.0333 (11)
C30	0.0873 (15)	0.0879 (16)	0.0690 (14)	0.0021 (13)	−0.0108 (11)	−0.0439 (12)
C31	0.167 (3)	0.0714 (15)	0.0735 (16)	−0.0217 (17)	−0.0086 (17)	−0.0416 (13)
C32	0.192 (3)	0.120 (2)	0.098 (2)	−0.093 (2)	0.033 (2)	−0.0632 (19)
C33	0.119 (2)	0.116 (2)	0.0828 (16)	−0.0573 (17)	0.0248 (14)	−0.0581 (15)

### Geometric parameters (Å, °)

**Table:**

O1—C2	1.3623 (19)	C19—C20	1.505 (2)
O1—C10	1.3772 (19)	C19—H19A	0.9700
C2—C3	1.336 (2)	C19—H19B	0.9700
C2—C11	1.468 (2)	C20—C21	1.375 (3)
C3—C4	1.442 (2)	C20—C25	1.384 (3)
C3—H3	0.9300	C21—C22	1.386 (3)
C4—O17	1.2287 (19)	C21—H21	0.9300
C4—C9	1.461 (2)	C22—C23	1.359 (3)
C5—O18	1.3521 (18)	C22—H22	0.9300
C5—C6	1.372 (2)	C23—C24	1.369 (4)
C5—C9	1.420 (2)	C23—H23	0.9300
C6—C7	1.395 (2)	C24—C25	1.386 (3)
C6—H6	0.9300	C24—H24	0.9300
C7—O26	1.3590 (19)	C25—H25	0.9300
C7—C8	1.378 (2)	O26—C27	1.4291 (19)
C8—C10	1.382 (2)	C27—C28	1.496 (2)
C8—H8	0.9300	C27—H27A	0.9700
C9—C10	1.388 (2)	C27—H27B	0.9700
C11—C16	1.376 (2)	C28—C33	1.365 (3)
C11—C12	1.381 (2)	C28—C29	1.371 (3)
C12—C13	1.376 (3)	C29—C30	1.373 (3)
C12—H12	0.9300	C29—H29	0.9300
C13—C14	1.364 (3)	C30—C31	1.361 (4)
C13—H13	0.9300	C30—H30	0.9300
C14—C15	1.362 (3)	C31—C32	1.367 (4)
C14—H14	0.9300	C31—H31	0.9300
C15—C16	1.373 (3)	C32—C33	1.378 (3)
C15—H15	0.9300	C32—H32	0.9300
C16—H16	0.9300	C33—H33	0.9300
O18—C19	1.415 (2)		
			
C2—O1—C10	119.99 (12)	O18—C19—H19A	110.1
C3—C2—O1	120.68 (15)	C20—C19—H19A	110.1
C3—C2—C11	127.46 (16)	O18—C19—H19B	110.1
O1—C2—C11	111.85 (14)	C20—C19—H19B	110.1
C2—C3—C4	123.57 (16)	H19A—C19—H19B	108.4
C2—C3—H3	118.2	C21—C20—C25	118.81 (18)
C4—C3—H3	118.2	C21—C20—C19	122.32 (17)
O17—C4—C3	121.05 (16)	C25—C20—C19	118.86 (19)
O17—C4—C9	124.67 (17)	C20—C21—C22	120.4 (2)
C3—C4—C9	114.27 (14)	C20—C21—H21	119.8
O18—C5—C6	123.46 (15)	C22—C21—H21	119.8
O18—C5—C9	115.42 (14)	C23—C22—C21	120.6 (2)
C6—C5—C9	121.11 (14)	C23—C22—H22	119.7
C5—C6—C7	119.92 (15)	C21—C22—H22	119.7
C5—C6—H6	120.0	C22—C23—C24	119.6 (2)
C7—C6—H6	120.0	C22—C23—H23	120.2
O26—C7—C8	124.22 (14)	C24—C23—H23	120.2
O26—C7—C6	114.59 (14)	C23—C24—C25	120.6 (2)
C8—C7—C6	121.18 (15)	C23—C24—H24	119.7
C7—C8—C10	117.46 (14)	C25—C24—H24	119.7
C7—C8—H8	121.3	C20—C25—C24	120.0 (2)
C10—C8—H8	121.3	C20—C25—H25	120.0
C10—C9—C5	115.96 (15)	C24—C25—H25	120.0
C10—C9—C4	119.12 (15)	C7—O26—C27	116.84 (13)
C5—C9—C4	124.91 (14)	O26—C27—C28	108.76 (14)
O1—C10—C8	113.76 (13)	O26—C27—H27A	109.9
O1—C10—C9	121.86 (14)	C28—C27—H27A	109.9
C8—C10—C9	124.36 (15)	O26—C27—H27B	109.9
C16—C11—C12	117.70 (17)	C28—C27—H27B	109.9
C16—C11—C2	120.75 (16)	H27A—C27—H27B	108.3
C12—C11—C2	121.55 (15)	C33—C28—C29	118.51 (19)
C13—C12—C11	120.97 (18)	C33—C28—C27	120.59 (19)
C13—C12—H12	119.5	C29—C28—C27	120.83 (19)
C11—C12—H12	119.5	C28—C29—C30	121.4 (2)
C14—C13—C12	120.5 (2)	C28—C29—H29	119.3
C14—C13—H13	119.8	C30—C29—H29	119.3
C12—C13—H13	119.8	C31—C30—C29	119.4 (2)
C15—C14—C13	119.0 (2)	C31—C30—H30	120.3
C15—C14—H14	120.5	C29—C30—H30	120.3
C13—C14—H14	120.5	C30—C31—C32	120.1 (2)
C14—C15—C16	121.0 (2)	C30—C31—H31	119.9
C14—C15—H15	119.5	C32—C31—H31	119.9
C16—C15—H15	119.5	C31—C32—C33	119.9 (3)
C15—C16—C11	120.8 (2)	C31—C32—H32	120.0
C15—C16—H16	119.6	C33—C32—H32	120.0
C11—C16—H16	119.6	C28—C33—C32	120.7 (2)
C5—O18—C19	119.10 (13)	C28—C33—H33	119.7
O18—C19—C20	107.99 (15)	C32—C33—H33	119.7
			
C10—O1—C2—C3	−5.7 (2)	C2—C11—C12—C13	−178.5 (2)
C10—O1—C2—C11	174.00 (14)	C11—C12—C13—C14	−0.4 (4)
O1—C2—C3—C4	0.4 (3)	C12—C13—C14—C15	−0.7 (4)
C11—C2—C3—C4	−179.18 (17)	C13—C14—C15—C16	1.1 (5)
C2—C3—C4—O17	−175.26 (19)	C14—C15—C16—C11	−0.4 (5)
C2—C3—C4—C9	5.6 (3)	C12—C11—C16—C15	−0.7 (4)
O18—C5—C6—C7	−178.38 (15)	C2—C11—C16—C15	178.9 (2)
C9—C5—C6—C7	0.2 (3)	C6—C5—O18—C19	6.3 (2)
C5—C6—C7—O26	179.15 (14)	C9—C5—O18—C19	−172.41 (14)
C5—C6—C7—C8	0.3 (3)	C5—O18—C19—C20	171.97 (14)
O26—C7—C8—C10	−178.51 (15)	O18—C19—C20—C21	−3.9 (2)
C6—C7—C8—C10	0.2 (3)	O18—C19—C20—C25	174.59 (16)
O18—C5—C9—C10	177.49 (14)	C25—C20—C21—C22	0.4 (3)
C6—C5—C9—C10	−1.2 (2)	C19—C20—C21—C22	178.92 (18)
O18—C5—C9—C4	−1.2 (2)	C20—C21—C22—C23	0.0 (3)
C6—C5—C9—C4	−179.92 (16)	C21—C22—C23—C24	−0.3 (4)
O17—C4—C9—C10	174.32 (18)	C22—C23—C24—C25	0.2 (4)
C3—C4—C9—C10	−6.5 (2)	C21—C20—C25—C24	−0.5 (3)
O17—C4—C9—C5	−7.0 (3)	C19—C20—C25—C24	−179.12 (19)
C3—C4—C9—C5	172.14 (16)	C23—C24—C25—C20	0.3 (4)
C2—O1—C10—C8	−174.37 (14)	C8—C7—O26—C27	3.3 (3)
C2—O1—C10—C9	4.4 (2)	C6—C7—O26—C27	−175.52 (15)
C7—C8—C10—O1	177.45 (14)	C7—O26—C27—C28	−179.56 (15)
C7—C8—C10—C9	−1.3 (3)	O26—C27—C28—C33	−114.1 (2)
C5—C9—C10—O1	−176.87 (14)	O26—C27—C28—C29	69.2 (2)
C4—C9—C10—O1	1.9 (2)	C33—C28—C29—C30	0.1 (3)
C5—C9—C10—C8	1.8 (2)	C27—C28—C29—C30	176.85 (18)
C4—C9—C10—C8	−179.40 (16)	C28—C29—C30—C31	−0.7 (3)
C3—C2—C11—C16	−5.0 (3)	C29—C30—C31—C32	0.5 (4)
O1—C2—C11—C16	175.35 (18)	C30—C31—C32—C33	0.3 (5)
C3—C2—C11—C12	174.54 (19)	C29—C28—C33—C32	0.7 (4)
O1—C2—C11—C12	−5.1 (2)	C27—C28—C33—C32	−176.1 (2)
C16—C11—C12—C13	1.1 (3)	C31—C32—C33—C28	−0.9 (5)

### Hydrogen-bond geometry (Å, °)

**Table:**

*D*—H···*A*	*D*—H	H···*A*	*D*···*A*	*D*—H···*A*
C12—H12···O1	0.93	2.37	2.701 (2)	101
C21—H21···O18	0.93	2.33	2.685 (2)	102
C16—H16···O17^i^	0.93	2.57	3.212 (3)	127
C30—H30···Cg1^ii^	0.93	3.12	3.838	135
C8—H8···Cg2^iii^	0.93	3.30	4.066	142
C27—H27B···Cg2^iii^	0.97	3.18	4.083	156

Symmetry codes: (i) −*x*+2, −*y*+1, −*z*; (ii) *x*, *y*, *z*+1; (iii) −*x*+2, −*y*+2, −*z*+1.
